# A Psychological Quarterly Retrospect

**Published:** 1856-01-01

**Authors:** 


### *A Psychological Quarterly Retrospect.*

[By the Editor.]

During the past quarter, two vacancies on the Board of Lunacy Commissioners have occurred; one occasioned by the retirement of Dr. Turner, and the second by the death of Mr. Mylne, the barrister. Dr. Turner's unfortunate infirmity of sight was considered materially to interfere with his usefulness and the efficient performance of his duties; and, consequently, it was considered necessary that he should vacate his official position, if not to a better man, at least to one who had a perfect use of his visual powers. Dr. Turner was much esteemed by all who had an opportunity of witnessing the unvarying gentlemanly urbanity, kindness, and tact with which he uniformly performed the duties assigned to him as one of the Medical Commissioners in Lunacy. He always exhibited great judgment in the exercise of his responsible functions; and whenever he had suggestions to make with reference to the domestic conduct of lunatic asylums, or the treatment of those confined in institutions licensed for the care and treatment of the insane, it was done with great gentlemanly discrimination, and with a kind and delicate consideration and regard for the feelings of others. Although exercising great authority, and armed with stringent legal powers, he had the good taste never to let others feel that they were in a position subordinate to himself. His loss will be, on this account, severely felt. His great experience in the investigation of cases of insanity much enhanced the value of his opinion on all occasions, and gave undeniable confidence in his judgment. It is understood that the Treasury has granted Dr. Turner a retiring pension of £350 per annum,—a small pittance considering his advanced age, and the lengthened period zealously devoted to anxious and important official services.

Mr. Mylne's death has caused a vacancy among the legal members of the board of the Commissioners. This gentleman's health had for some time given his family and friends cause for much anxiety. He suffered from great bronchial irritation, of which he is reported ultimately to have died at his residence in Onslow Square. One vacancy has been filled up by the appointment of Dr. Wilkes, for many years Medical Superintendent of the Stafford County Lunatic Asylum; and Mr. Lutwidge, who for a considerable period has efficiently occupied the post of

Secretary to the Board of Commissioners in Lunacy, is appointed successor to Mr. Mylne. Dr. Wilkes brings to the performance of his duties a large amount of practical experience. He was appointed, within the last twelve months, one of the Commissioners to visit and report on the condition of Irish Lunatic Asylums; and in the performance of this duty he is said to have exhibited great business and administrative talent. Dr. Wilkes's antecedents are all in his favour: he will undoubtedly earn fresh laurels in his new sphere of usefulness. Mr. Lutwidge for many years acted as a commissioner in lunacy under a former statute, having been appointed to that post by Lord Chancellor Lyndhurst. He, therefore, possesses the recommendation of experience. His appointment will give unqualified satisfaction to the profession as well as the public. He is an active, accomplished, and sagacious lawyer, and will resume his post of Visiting Commissioner with a conscientious determination to discharge the duties devolving upon him with judicious zeal.

The nomination of Dr. Wilkes to the Medical Commissionership has created a vacancy of Medical Superintendent at the Stafford County Asylum. The post is a lucrative one, the salary being £700 per annum.

The subject of intemperance, *as a type of disease* requiring legal interference, has frequently been a topic of discussion in the "Psychological Journal." This question has recently been litigated in the High Court of Justiciary in Edinburgh. We extract from a local paper the following report of the proceedings, which will, no doubt, prove of interest to the English public :—

"The Court met to-day, December 8, on the rising of the Second Division, to consider the note of suspension and liberation, previously before them in preliminary stages, of Mr. Giles Gerard—lately residing in Elgin, and now a prisoner in Morningside Asylum—against Mr. William Grigor, Procurator-Fiscal of Elgin. The complainant—a gentleman of independent fortune—had been examined by the Sheriff Substitute of Elgin, under the Lunacy Act, and, on the evidence of his wife, his medical attendant, and others, been found by the sheriff to be 'furious and fatuous, or a lunatic, and in a state threatening danger to the lieges,' and was by him committed to a lunatic asylum, which it was eventually arranged should be the asylum at Morningside. From the evidence taken before the sheriff, it appeared that Mr. Gerard was subject to frequent fits of furiosity; arising, it was said, among other causes, from intemperance, and in this condition was dangerous to his family. The complainer craved liberation on the plea principally of wrongous detention and sanity, and subsidiarily on several grounds of informality. He adduced the joint certificate of Dr. Christison and Dr. Combe, that they were unable to discover any indications of insanity, and believed him to be at the present time sane. A certificate was produced, on the other hand, from the physicians of the asylum, stating they had not, during his brief residence there, been able to satisfy themselves that he was of perfectly sound mind, or capable of so regulating his conduct as not to be dangerous either to himself or others. The principal question argued on Saturday was the competency of the sheriff's judgment. Mr. Logan and Mr. Young appeared for the complainer; the Dean of Faculty and Mr. A. R. Clark for the respondent; and Mr. Andrew Mure for Mrs. Gerard. After hearing the case, the Court were divided in opinion. The Lord Justice-Clerk, Lord Cowan, and Lord Deas, were of opinion it was sufficient in the case that the sheriff should be satisfied, and that, therefore, his judgment was perfectly correct. Without deciding that point, or reviewing his decision, they also thought that, upon the evidence, it was a right one. What the result of throwing further light on the complainer's past and present state might be, their lordships did not anticipate, but this detention was not necessarily a permanent one; it being only 'until cured.' It was unnecessary, on the other hand, that the furiosity should be continuous; for occasional and intermittent bursts of furiosity on the part of a person, at other moments sane, might be sufficient to warrant such proceedings; while the danger to his family, from these fits, entitled them to this protection. Lords Handyside and Ardmillan took a different view. Ascribing the furiosity to intemperance, they thought the application of the Lunacy Act an extreme and unusual remedy. It was proved, said Lord Ardmillan, that the complainer was a violent man; but it was not proved he was a madman. It was no proof that a man was mad, because, when drunk, he did violent or furious things. That might make him a fit subject for police restraint, but it did not justify his being shut up as a lunatic. Many thousand husbands, perhaps, were drunk in Glasgow every Saturday night, and a large proportion of them probably were violent, but were they to crowd their lunatic asylums with these men? The Court, by a majority, thus sustained the sheriff's decision. The case, however, will come up again, on an application by Mr. Gerard's counsel to have a fuller inquiry into his present and past condition."

There has been much gossip in the literary and scientific world relative to some *Spirit Rapping* experiments, alleged to have been performed at the private residence of Mr. Rymer, a London solicitor, at Ealing, in the presence of Lord Brougham, Sir David Brewster, Mr. Hume, and Mrs. Trollope. All these distinguished individuals are reported to have become converts to the spiritual phenomena. Sir David Brewster has, however, since deemed it necessary to repudiate the fact of his conversion. Lord Brougham and Mrs. Trollope have not spoken out on the subject. Sir David Brewster's letter will be read with interest, and deserves to be placed permanently on record. He writes,—

"It is true that I saw at Cox's Hotel, in company with Lord Brougham, and at Ealing, in company with Mrs. Trollope, several mechanical effects which I was unable to explain. But though I could not account for all these effects, I never thought of ascribing them to spirits stalking beneath the drapery of the table; and I saw enough to satisfy myself that they could all be produced by human hands and feet, and to prove to others that some of them, at least, had such an origin. Were Mr. Hume (the American medium) to assume the character of the Wizard of the West, I would enjoy his exhibition as much as that of other conjurers; but when he pretends to possess the power of introducing among the feet of his audience the spirits of the dead, of bringing them into physical communication with their dearest relatives, and of revealing the secrets of the grave, he insults religion and common sense, and tampers with the most sacred feelings of his victims." In another letter Sir David enters in more detail into what Lord Brougham and he saw done by "the spirits," and what they did *not* see: "It is not true that the accordion *played an air throughout* in Lord Brougham's hands. It merely squeaked. It is not true, as stated in an article referred to by Mr. Hume, that Lord Brougham's 'watch was taken out of his pocket, and found in the hands of some other person in the room.' No such experiment was tried. . . . . At Mr. Cox's house, Mr. Hume, Mr. Cox, Lord Brougham, and myself sat down to a small table, Mr. Hume having previously requested us to examine if there was any machinery about his person, an examination, however, which we declined to make. When all our hands were upon the table noises were heard—rappings in abundance; and, finally, when we rose up the table actually rose, as appeared to me, from the ground. . . . . Besides the experiments with the accordion, already mentioned, a small hand-bell to be rung by the spirits, was placed on the ground, near my feet. I placed my feet round it in the form of an angle, to catch any intrusive apparatus. The bell did not ring; but when taken to a place near Mr. Hume's feet, it speedily came across and placed its handle in my hand. This was amusing. It did the same thing bunglingly to Lord Brougham, by knocking itself against his lordship's knuckles, and after a jingle it fell. The *séance* was most curious at Ealing, where I was a more watchful and a more successful observer. I will not repeat the revelations made to Mrs. Trollope, who was there, lest I should wound the feelings of one so accomplished and sensible. I remember them with unmingled pain. The spirits were here very active, prolific in raps of various intonations, making long tables heavy or light at command; tickling knees, male and female, but always on the side next the medium; tying knots in handkerchiefs drawn down from the table, and afterwards tossed upon it; and prompting Mr. Hume, when he had thrown himself into a trance, to a miserable paraphrase on the Lord's Prayer. During these experiments I made some observations worthy of notice. On one occasion, the Spirit gave a strong affirmative answer to a question by *three raps*, unusually loud. They proceeded from a part of the table exactly within the reach of Mr. Hume's foot, and I distinctly saw three movements in his loins, perfectly simultaneous with the three raps."

We have to record among the deaths the decease of Dr. T. Romeyn Beck, one of the able and active managers of the *New York State Lunatic Asylum*. The readers of the "American Journal of Insanity" are, no doubt, familiar with his name. He took a deep interest in the success of our accomplished American contemporary, and watched with great care the progress of the cases under treatment in the State Asylum previously referred to. The following eloquent eulogium on Dr. Beck is copied from the *Albany Evening Journal* of November 19th:—

"Dr. Beck's health had been gradually declining for several months. In the absence of any organic disease, hopes of his recovery were entertained until some few weeks ago, when an unfavourable opinion was obtained from high medical authority. Since that period his family and friends, prepared for the worst, have awaited an event which bereaves them and the community of a man who in all things was the type and exemplar of his race.

"Dr. Beck's mission was one of practical usefulness. During the quarter of a century that he devoted himself laboriously to the instruction of youth, as the principal of our academy, people wondered how a man so gifted could content himself with a position so comparatively humble. The answer is, that Dr. Beck was unselfish and unambitious. He loved his school, his friends, his associates, and above all his home. These were, to him, sources of happiness too precious to be sacrificed. He pursued, therefore, with diligence and cheerfulness, the 'even tenor of his way,' raising up generation after generation of thoroughly educated young men, whose first duty and highest privilege through life has been to acknowledge, with grateful hearts, obligations to their beloved preceptor.

"Dr. Beck aimed to render all his scientific and literary acquirements available. His knowledge was held in trust for the benefit of others. His mind, like a tree upon a common, bore fruit for the community. He was a man of simple manners, genial nature, social habits, large humanity, and radiant faith. Almost half a century was passed among us in the active discharge of responsible public duties. His efforts to promote education, science, improvement, virtue, and Christianity, were always well and wisely directed.

"Dr. Beck's associations through life have been with the truly good and great. His society was sought by all who appreciated public worth and social excellence. Those who for so many years enjoyed both in their daily intercourse with him, while deploring his loss will cherish his memory. But to other hearts—hearts with which his own was intertwined—the bereavement comes with a crushing weight. In the halls his presence brightened and gladdened, there is now darkness and sorrow."

The christian and enlightened sentiments of a large body of American citizens have been outraged by the barbarous, cruel, and unjustifiable execution at Alexandria of a boy only ten years of age! It is indeed a sad and painful duty to have to record so horrible and lamentable a catastrophe. A New Orleans paper thus speaks of the execution of this unhappy and irresponsible youth:—

"The execution of a boy named Frank, for the murder of Rev. J. J. Weems, took place in the United States lately. It is strange to say that the majority of the citizens of Alexandria, and, in fact, the inhabitants all round, were anxious to see him executed; though on the fatal day, when it came to pass, there were not a dozen people there. Some rode forty miles to witness this painful drama, but he was executed and buried by the time they reached Alexandria. On the day before he was called to face death, some gentlemen visited him and propounded questions to him; but his answers were and could be no other than childish. He was only ten years old. The gentlemen told him the sheriff was to hang him on the next morning, and asked him what he thought of it, and whether he had made his peace with God, and why he did not pray? His answer was, 'I have been hung many a time!' He was, at the time, amusing himself with some marbles he had in his cell. He was playing all the time in jail, never once thinking that death was soon to claim him as his victim. To show you how a child's mind ranges when about to die, I will mention that, when upon the scaffold, he begged to be permitted to pray, which was granted, and then he commenced to cry. O what a horrible sight it was!"

Our readers will recollect that we published in the "Psychological Journal," during the preceding year, an elaborate extract from one of the Boston medical journals relative to certain hallucinations alleged to be induced by chloroform. The observations referred to sprang out of the case of Mr. Beale, an American dentist, who was accused of having acted with extreme indelicacy and impropriety towards a female patient to whom he had exhibited chloroform preparatory to performing some trifling operation in dental surgery. The patient in question, after recovering from the effects of chloroform, charged Mr. Beale with having committed some serious professional indiscretion. Mr. Beale was publicly brought to trial, found guilty, and sentenced to prolonged imprisonment. It appears from the subjoined extract from the *Philadelphia Ledger*, that this gentleman has received a free pardon. We are pleased to have it in our power to announce this gratifying fact, for the evidence of his guilt was anything but conclusive.

"Governor Pollock has extended his clemency to Dr. Beale, and remitted the remainder of his sentence of imprisonment, which was four years and six months, beginning on the 28th of November, 1854. He has served, therefore, about one year of his term. The pardon states the reasons which induced the Governor to extend this favour.

"He had received communications from about one hundred and forty dentists and twenty-three physicians of this city and the country, stating their belief that testimony as to matters transpiring under the influence of ether is unsafe and unreliable; from a number of other physicians named, that they believe him innocent; from a large number of the bar and citizens of various States, including the names of Governors, Attorneys-General, &c., that they believe he was convicted on insufficient testimony; from a number of clergymen, that they believe him innocent; from the Mayor of Philadelphia and fifty members of the Philadelphia City Councils; from members of the Legislature, Judges of the Supreme Court, editors of Philadelphia newspapers, and five thousand other citizens of Pennsylvania and New York, with five of the jury on the trial, all asking for his pardon. After enumerating all these facts, the Governor says:—

"And whereas the Board of Inspectors of the said Philadelphia County Prison (as appears by their communication on file in the office of the Secretary of the Commonwealth) have unanimously recommended the pardon of the said Dr. Stephen T. Beale, because, in their opinion, the end contemplated by the law in the moral reform of the prisoner has been attained—because full and ample satisfaction has been rendered to public sentiment by the imprisonment he has already undergone—because his health is undoubtedly breaking down under the sufferings of body and mind which he has already endured; and because the destitute condition of his aged parents and bereaved and sorrowing wife and children imperatively demand the presence and support of their son, husband, and father.

"And whereas, after a full and careful examination of the facts and evidence in the case, aided by the scientific discussions to which it has given rise, (without any intention to reflect upon the prosecutrix, who no doubt testified to what she believed did occur—nor to impugn the integrity of the learned Judge who tried the case—nor the honesty of the Jury who convicted the prisoner,) *I am now satisfied* that the defendant, Dr. Stephen T. Beale, is *not guilty* of the crime whereof he stands charged, and was convicted upon evidence unreliable in its character and insufficient in amount.

"I do, therefore, in consideration of the premises, pardon the said Dr. Stephen T. Beale of the crime whereof he is convicted as aforesaid, and he is hereby fully pardoned accordingly."

Mr. G. W. Lewes' charming Life of the great and illustrious Goethe has formed the subject of general conversation and criticism in all the literary circles of the metropolis. We quite agree with the writer of an analysis of the work that appeared in No. VI. of the *Saturday Review*, that few Englishmen were better fitted to be the biographer of Goethe than Mr. Lewes. His deeply interesting work will find a permanent position among the standard British classics. It is written with great good taste and feeling, with an elevated and correct appreciation of the character of the great German dramatic poet and novelist, a profound acquaintance with the philosophy with which that eminent man was so richly imbued, and which gave tone, character, and dignity to the eventful epoch in which he flourished. We refer to Mr. Lewes' able production for the purpose of quoting his graphic account of Goethe's singularly affecting and touching death. It has a deep psychological import:—

"It was the 22nd March, 1832, he tried to walk a little up and down the room, but after a turn, he found himself too feeble to continue. Reseating himself in the easy chair, he chatted cheerfully with Ottilie on the approaching Spring, which would be sure to restore him. He had no idea of his end being so near.

"The name of Ottilie was frequently on his lips. She sat beside him, holding his hand in both of hers. It was now observed that his thoughts began to wander incoherently. 'See,' he exclaimed, 'the lovely woman's head—with black curls—in splendid colours—a dark background!' Presently he saw a piece of paper on the floor, and asked them how they could leave Schiller's letters so carelessly lying about. Then he slept softly, and on awakening, asked for the sketches he had just seen—the sketches of his dream. In silent anguish they awaited the close now so surely approaching. His speech was becoming less and less distinct. The last words audible were: *More light!* The final darkness grew apace, and he, whose eternal longings had been for more Light, gave a parting cry for it, as he was passing under the shadow of Death.

"He continued to express himself by signs, drawing letters with his forefinger in the air, while he had strength, and finally, as life ebbed, drawing figures slowly on the shawl which covered his legs. At half-past twelve he composed himself in the corner of the chair. The watcher placed a finger on her lip to intimate that he was asleep. If sleep it was, it was a sleep in which a life glided from the world. He woke no more.

"So died," says the eloquent writer of the analysis to which we have referred, "Germany's greatest son—great as a dramatist—great as a novelist, holding no mean rank in physical science, pre-eminently great as a lyric and idyllic poet, great as a man, were it but for the wonderful variety of his gifts and the harmony of his intellectual powers; but greatest of all because of his noble charity, his large, loving, kind heart. To admit that he had errors and weakness withal, is but to admit that he was a man. In youth, especially, his ardent and impressionable temperament made him at times fickle, selfish, and cruel, to those that loved him. At a later period, his conduct in regard to Christiane Vulpius, gave just offence and scandal to his countrymen; but we must not forget that he did afterwards all that could be done to repair the sin and blot out the shame. We may regret not to find in his writings and his conversation more explicit recognition of the doctrines of our common Christian faith; but, take him all in all, few men have lived lives so great, so good as his. At any rate, it does not belong to us to judge him. One day, we too, like him, shall have 'MORE LIGHT.'"*

In our *résumé* of the literature of the quarter, we first proceed to a consideration of Mr. Charles Dickens' new serial publication "Little Dorrit." This new picture of English life and character is susceptible

* "The Saturday Review," No. 6.

of a psychological interpretation. Mr. Dickens will be doing a great national service if he succeed in *popularizing* among the working classes the Sabbath day, and enshrouding this holy festival of the church with a halo of sunny cheerfulness. In Mrs. Clennam, he endeavours to delineate the hard, rigorous puritan; a Mawworm, "stern of face and unrelenting of heart, making religion a weapon of defence, and a pretext for the indulgence of tyranny." Mrs. Clennam is considered as a *type* of pseudo-religionists who scowl, shake their heads, groan and look unutterable things if a smile is but permitted to play upon the countenance during the day set apart for devotional exercises. God never designed the pure worship of Himself to be synonymous with gloom, melancholy, despondency, and insanity. Bishop South says, "to be religious, it is not necessary to be dull." And Dr. Watts still more emphatically declares, that

> "Religion never was designed
> To make our pleasures less."

It will be a source of satisfaction to many that Mr. Dickens is directing his great talents to the consideration of this important social question. No man exercises, for good or for evil, so overwhelming an influence upon the national mind and character. "To whom much is given, much will be required." We have no fear of Mr. Dickens stamping by the authority of his great name anything that has not a moral tendency; and although he is now travelling upon slippery, dangerous, and delicate ground, and battling with principles deeply rooted in the human heart—we have no misgivings or apprehension for the result. Such a subject in less gifted and injudicious hands might be productive of serious mischief; but we feel assured that Mr. Dickens will never forget that he is dealing with a topic fraught with the deepest interest to the eternal welfare of the human race; and, with a right regard for what he may conceive to be the prejudices, as well as the failings, foibles, and weaknesses of others, he will, by exposing hollow-hearted, selfish hypocrisy, hugging and protecting itself under the cloak of a false and affected sanctity, rid the religion of our common Saviour, and the christianity of the Bible, of much that now tends to impede its practical usefulness, and to obscure its bright and cheerful effulgence.

Mr. Dickens thus describes an English gloomy Sunday evening:—

"It was a Sunday evening in London, gloomy, close, and stale. Maddening church bells of all degrees of dissonance, sharp and flat, cracked and clear, fast and slow, made the brick and mortar echoes hideous. Melancholy streets in a penitential garb of soot, steeped the souls of the people who were condemned to look at them out of windows in dire despondency. In every thoroughfare, up almost every alley, and down almost every turning, some doleful bell was throbbing, jerking, tolling, as if the plague were in the city and the dead-carts were going round. Everything was bolted and barred that could by possibility furnish relief to an overworked people. No pictures, no unfamiliar animals, no rare plants or flowers, no natural or artificial wonders of the ancient world—all *taboo* with that enlightened strictness, that the ugly South Sea gods in the British Museum might have supposed themselves at home again. Nothing to see but streets, streets, streets. Nothing to breathe but streets, street, streets. Nothing to change the brooding mind, or raise it up. Nothing for the spent toiler to do, but to compare the monotony of his seventh day with the monotony of his six days, think what a weary life he led, and make the best of it—the worst, according to the probabilities."

To the poor half-starved, weary, anxious, heart-broken, and over-worked mechanic who is compelled, in order to preserve a decent position amongst his fellow men, to devote twelve or fifteen hours out of the twenty-four for six days in the week, to hard and unceasing toil in an atmosphere redolent of odious, offensive, and noxious particles, the Sunday as delineated by Dickens may not engender feelings of a pleasurable or religious character. We deeply regret that such should be the case. It is to be lamented that the seventh day of the week is not emphatically to the working population a day of *bonâ fide* rest. It would be so if by common consent some other day or even part of a day were set aside as a national holiday for recreation, pleasure, and health, and the Sabbath day be properly viewed as one that ought to be devoted to religious exercises. The mind requires relaxation and amusement. Without a certain amount of pleasurable excitement, there cannot exist a healthy state of intellect or moral feeling. This is practicable without any desecration of the Sabbath day. Mr. Dickens continues:—

"At such a happy time, so propitious to the interests of religion and morality, Mr. Arthur Clennam, newly arrived from Marseilles by way of Dover, and by Dover coach the Blue-eyed Maid, sat in the window of a coffee-house on Ludgate-hill. Ten thousand responsible houses surrounded him, frowning as heavily on the streets they composed as if they were every one inhabited by the ten young men of the Calender's story, who blackened their faces and bemoaned their miseries every night. Fifty thousand lairs surrounded him where people lived so unwholesomely, that fair water put into their crowded rooms on Saturday night, would be corrupt on Sunday morning; albeit my lord, their county member, was amazed that they failed to sleep in company with their butcher's meat. Miles of close wells and pits of houses, where the inhabitants gasped for air, stretched far away towards every point of the compass. Through the heart of the town a deadly sewer ebbed and flowed, in the place of a fine fresh river. What secular want could the million or so of human beings whose daily labour, six days in the week, lay among these Arcadian objects, from the sweet sameness of which they had no escape between the cradle and the grave—what secular want could they possibly have upon their seventh day? Clearly they could want nothing but a stringent policeman.

"Mr. Arthur Clennam sat in the window of the coffee-house on Ludgate-hill, counting one of the neighbouring bells, making sentences and burdens of songs out of it in spite of himself, and wondering how many sick people it might be the death of in the course of the year. As the hour approached, its changes of measure made it more and more exasperating. At a quarter, it went off into a condition of deadly lively importunity, urging the populace in a voluble manner to Come to Church, Come to Church, Come to Church! At the ten minutes, it became aware that the congregation would be scanty, and slowly hammered out in low spirits, They *wont* come, they *wont* come, they *wont* come! At the five minutes, it abandoned hope, and shook every house in the neighbourhood for three hundred seconds, with one dismal swing per second, as a groan of despair."

To minds properly imbued with religious feelings, and with hearts attuned to sacred melody, the church bells calling the population together to join in the worship of God ought not to excite any but elevating and pleasurable emotions.

"There is in souls a sympathy with sounds,
And as the mind is pitch'd, the ear is pleased;
With melting airs, or martial, brisk, or grave.
Some chord in unison with what we hear
Is touch'd within us, and the heart replies.
How soft the music of these village bells
Falling at intervals upon the ear
In cadence sweet! now dying all away,
Now pealing loud again, and louder still;
Clear and sonorous as the gale comes on
With easy force it opens all the cells
Where mem'ry slept."

There is a charming and soothing music in the merry chimes of church bells, as they echo through the copse, and are wafted across the flowery mead, and float along the village green. How often do they on a quiet Sabbath morn fall with sweetness upon the ear, as their peals encircle the neighbouring hills, or joyously rush down into the woodland dale, sounding like the revelry of friends:—

"So have I stood alone on Isis bank,
To hear the merry Christchurch bells rejoice;
So have I sat, too, in thy honour'd shades,
Distinguished Magdalen on Cherwell's banks,
To hear thy silver Wolsey tones so sweet," &c.

The church bells, whether in town or country, *should* speak of LOVE, of HOPE, of PEACE, of CHARITY: of FORGIVENESS OF SINS; of GOODWILL TOWARD MEN; of REST—eternal and happy REST—to the weary traveller, when he arrives at the termination of his sad earthly pilgrimage.

"'Thank Heaven!'" said Clennam, when the hour struck, and the bell stopped.

"But its sound had revived a long train of miserable Sundays, and the procession would not stop with the bell, but continued to march on. 'Heaven forgive me,' said he, 'and those who trained me. How I have hated this day!'

"There was the dreary Sunday of his childhood, when he sat with his hands before him, scared out of his senses by a horrible tract which commenced business with the poor child by asking him in its title, why he was going to perdition?—a piece of curiosity that he really in a frock and drawers was not in a condition to satisfy . . . . . There was the sleepy Sunday of his boyhood, when, like a military deserter, he was marched to chapel by a picquet of teachers three times a day, morally handcuffed to another boy; and when he would willingly have bartered two meals of indigestible sermon for another ounce or two of inferior mutton at his scanty dinner in the flesh. There was the interminable Sunday of his nonage; when his mother, stern of face and unrelenting of heart, would sit all day behind a Bible—bound like her own construction of it in the hardest, barest, and straightest boards, with one dinted ornament on the cover like the drag of a chain, and a wrathful sprinkling of red upon the edges of the leaves—as if it, of all books! were a fortification against sweetness of temper, natural affection, and gentle intercourse. There was the resentful Sunday of a little later, when he sat glowering and glooming through the tardy length of the day, with a sullen sense of injury in his heart, and no more real knowledge of the beneficent history of the New Testament, than if he had been bred among idolaters. There was a legion of Sundays, all days of unserviceable bitterness and mortification, slowly passiug before him."

The poor child leaves his home, and does not return until he is a man. How graphic is Mr. Dickens's account of Mr. Arthur Clennam's interview with his mother!

"Arthur followed him up the staircase, which was panelled off into spaces like so many mourning tablets, into a dim bedchamber, the floor of which had gradually so sunk and settled, that the fireplace was in a dell. On a black bier-like sofa in this hollow, propped up behind with one great angular black bolster, like the block at a state execution in the good old times, sat his mother in a widow's dress.

"She and his father had been at variance from his earliest remembrance. To sit speechless himself in the midst of rigid silence, glancing in dread from the one averted face to the other, had been the peacefullest occupation of his childhood. She gave him one glassy kiss, and four stiff fingers muffled in worsted. This embrace concluded, he sat down on the opposite side of her little table. There was a fire in the grate, as there had been night and day for fifteen years. There was a kettle on the hob, as there had been night and day for fifteen years. There was a little mound of damped ashes on the top of the fire, and another little mound swept together under the grate, as there had been night and day for fifteen years. There was a smell of black dye in the airless room, which the fire had been drawing out of the crape and stuff of the widow's dress for fifteen months, and out of the bier-like sofa for fifteen years.

"'Mother, this is a change from your old active habits.'

"'The world has narrowed to these dimensions, Arthur,' she replied, glancing round the room. 'It is well for me that I never set my heart upon its hollow vanities.'

"The old influence of her presence and her stern strong voice, so gathered about her son, that he felt conscious of a renewal of the timid chill and reserve of his childhood."

How much insanity, what an amount of miserable and incurable religious thought, we have known to spring out of such asceticism. It is a frightful cause of mental derangement, nervous and hysterical disorder. In the wards of our lunatic asylums, private as well as public, may be seen many sad cases of this type. Gloomy, false, and misanthropic views of religion, and mistaken notions of man's relation to his great and wise Creator, are, alas! frequent causes of mental alienation in its most distressing and incurable form. Mr. Dickens's "Little Dorrit" will conduce greatly to the public advantage, by rousing our attention to this subject, as he has in his former writings enlisted our sympathies in behalf of our over-worked and half-starved population. The picture which the poet Massey draws of his own early career is a truthful description of what thousands of our fellow creatures have to encounter in early life in our manufacturing districts.

"Born in a little house, the roof of which no man could stand upright under; at eight years of age earning his meagre living in the adjacent silk mills; rising at five, and toiling there till half-past six in the evening; seeing the sun only through factory windows, breathing an atmosphere laden with oily vapour;—what a life for a child!" The mill is burnt down, and the children hold a jubilee (and who can wonder?) over its blazing ruins.

"I have had no childhood," says Gerald Massey. "Ever since I can remember, I have had the aching fear of want throbbing in heart and brow. The child comes into the world like a new coin with the stamp of God upon it; and in like manner as sovereigns are sweated down by hustling them in a bag to get gold dust out of them, so is the poor man's child hustled and sweated down in this bag of society to get wealth out of it."

The portion of our population who unhappily breathe God's glorious air under circumstances so graphically pourtrayed by one who thus touchingly records

"The short, but simple annals of the poor,"

enter the world with a stern and unalterable conviction that the rich and aristocratic classes of society have no kind, generous, and yearning impulses towards their poorer brethren. We regret to say that such is the *general* impression among those who form the bone, the muscle, and sinews of the population of this country. The writings, the speeches, the poetry, the songs of the working classes constitute an embodiment of this idea. Take, for instance, Mr. Noel's affecting description of the "Pauper's Drive," a poem only second to Hood's immortal "Song of the Shirt," and the "Bridge of Sighs."

There's a grim one-horse hearse in a jolly round trot,
To the churchyard a pauper is going, I wot;
The road it is rough, and the hearse has no springs,
And hark to the dirge that the sad driver sings:
Rattle his bones over the stones;
He's only a pauper whom nobody owns.

Oh, where are the mourners? alas, there are none!
He has left not a gap in the world now he's gone—
Not a tear in the eye of child, woman, or man:
To the grave with his carcase as fast as you can.
Rattle, &c.

What a jolting, and creaking, and splashing, and din!
The whip, how it cracks—and the wheels, how they spin!
How the dirt right and left o'er the hedges is hurled!
The pauper at length makes a noise in the world.
Rattle, &c.

Poor pauper defunct! he has made some approach
To gentility now that he's stretched in a coach:
He's taking a drive in his carriage at last,
But it will not be long if he goes on so fast.
Rattle, &c.

But a truce to this strain, for my soul it is sad
To think that a heart in humanity clad
Should make, like the brutes, such a desolate end,
And depart from the light without leaving a friend.
Rattle his bones over the stones;
Though a pauper, he's one WHOM HIS MAKER YET OWNS.

Mr. Samuel Baily's "Letters on the Philosophy of the Human Mind," will not detract from this gentleman's well-earned reputation as a liberal and profound thinker and an elegant and accomplished writer. Mr. Baily's "Letters" contain no novel or striking metaphysical views; but the principles as taught by Reid and Stewart are lucidly enunciated. Karl Fortlage's "System der Psychologie" is exciting some notice in Germany. Fortlage is already favourably known by his countrymen for his work, entitled "Genetische Geschichte der Philosophie seit Kant." In speaking of the "System der Psychologie," an able contemporary remarks, that the point of view from which the author presents psychology is the experimental. He totally rejects the speculative *à priori* method as having been the bane of this subject, and as having, not unnaturally, created an opinion of the uncertainty of all mental philosophy—an opinion really as unfounded as that of antiquity on the uncertainty of natural philosophy. In an able criticism of this work that appeared in the "Westminster Review," the writer observes:—

"But in establishing psychology as an empirical science, we are not to resolve it as has been sometimes done into a branch of the inductive knowledge of nature—a physiology of nervous process. This is to confound two distinct spheres of observation—the inner and the outer. The outer is observed by the senses, and is the sphere of the natural sciences; the inner is observed by the inner sense or consciousness. This determination of the peculiar field of observation forms the starting point which discriminates this method from the speculative, which starts from an *à priori* conception of the nature of physical life. But though we discard the speculative, and take our stand on observation, yet we must have a scientific element in our psychology, which would be otherwise a mere record of phenomena. The merely empirical psychologist would resemble an anatomist who should fancy that in anatomy he possessed a complete physiology. The experimentalists hitherto have treated the mind as a sort of 'camera obscura,' into which we had but to look and photograph what went on there. In this way empirical psychology has fallen as much short of a science of mind as the speculatists had overleapt it. The first hopes for a better era of mental science began with the double method or the union of the speculative with the empirical. Of this double method there are two types. The first that of which Herbert is the representative. In this certain speculative laws—any that have probability in their favour—are assumed for the purpose of serving as a clue through the chaos of observations. But with this difference from the old purely speculative method, that the observation is not employed to prove assumed laws, but the principles are used to aid in collating the observations. The other form of the double method is represented by Beneke. This seeks in observation itself for any empirical laws which it can apparently establish, and having got such, it erects them into universal laws of mind. This latter method is obviously that which has approached nearest to the true method of psychological study. It has the defect of jumping too abruptly from the phenomenological to the ætiological. But Beneke is, after all, the predecessor with whom the author considers his own labours to be in the closest connexion. We must not, however, in condemning this method, overlook the great services rendered to the science by the sensualistic psychologist of the old school, now entirely passed away. They brought various phenomena to light, while the speculative schools have done nothing but encumber the ground with empty theories. Besides this general relation to Beneke, Professor Fortlage adopts a principle from Reinhold, Freudelenberg, and Schopenhauer, respectively. From Reinhold—the active ingredient in sensuous perception, which furnishes the link between the *à priori* and *à posteriori* so much wanted in the Kantian system; and the Schopenhauer—the will as underlying consciousness, or forming the substratum of all psychical existence.

"The present 'first part' contains, besides an introduction and statement of the view point, four chapters; 1. On the Consciousness; 2. On the General, 3. On the Particular Properties of the Contents of a Presentation; 4. On the relation of the Consciousness to the contents of a Presentation."

Dr. Noble has brought out a new and more elaborate edition of his "Elements of Psychological Medicine." He has re-written several portions of his treatise. It is a creditable production, and should be read by all psychological students. The work is what Dr. Noble purports it to be—namely, an elementary essay; but nevertheless it may be read with profit by all interested in the subject of medical psychology. We direct attention to Dr. Noble's labours in this part of our journal, as want of space will prevent the publication in this number of the full analysis of his views, which we had prepared for the first number of our new series.

Dr. Hume Williams's work on "Mental Unsoundness in its Medical and Legal Relations," is one of the most important psychological productions of the day. We have no treatise on the subject in the English language to equal it in interest and value. Dr. Williams's style is vigorous, terse, classical, and lucid. He is evidently master of his subject. He develops in his work principles of judicial psychology of the deepest importance to the administration of justice in cases involving the existence of insanity, and to all questions of a civil and criminal responsibility relating to the insane. We intend to return to this essay in a future number.

We must not omit to refer to Dr. Steinthal's "Logik, Grammatik, u. Psychologie, ihre Principien und ihr Varhaltness zu einander."* It consists in a violent attack upon Wilhelm Von Humboldt and the Beckerians, MM. Muller and Aufrecht, for asserting "that the philosophy of language has now attained its maturity." Dr. Steinthal asserts that

* Berlin, 1855.

these authorities neither feel or comprehend the necessity for such a philosophy, and still less have the means of satisfying such necessity.

Mr. Bain's "Senses and the Intellect"* has come in for its full share of critical analysis and commendation. It is an admirable metaphysical essay, and may, it is said, be compared with the best productions of German industry, and deserves a place by the side of Mill's "Treatise on Logic." Mr. Bain advocates a closer relationship between psychology and the higher branches of physiology than has hitherto been deemed necessary. He considers it indispensable that the psychologist should be well versed in the higher departments of anatomy, as well as special physiology,

Dr. Ferrier's "Institutes of Metaphysic" demands more than a passing notice. It is an able and philosophical essay. His attempt to bring the great subject of metaphysics within the range of ordinary comprehension, and to simplify the most abstruse and subtle of human investigations, deserves our warmest praise. There is a freshness and originality about the work, which strongly commends it to the reader's attention.

An essay on the "Philosophy of the Infinite, with special reference to the theories of Sir W. Hamilton and M. Cousin," by Mr. H. Calderwood, has excited some attention among metaphysicians. It requires no little moral courage to break a lance with such acute metaphysicians as Sir W. Hamilton and M. Cousin. Mr. Calderwood's essay is said to be, "to a degree almost laughable, a repetition of Sir W. Hamilton;" using the arguments, and even the identical language of the great Edinburgh metaphysician. But we must reserve any further criticism of this, and Mr. Spencer's valuable work on the "Principles of Psychology," for our next number.

The late remarkable and mysterious deaths of Dr. Franck and his son at the Albion Hotel, Brighton, cannot be passed over in silence. The question involved is one deeply interesting to the physician engaged in the study of judicial psychology. We subjoin an epitome of the facts illustrative of this deeply affecting tragedy:—

Dr. Herman Franck, a gentleman about fifty years of age, arrived at Brighton, from Gosport, accompanied by his son Hugo, a fine lad of seventeen or eighteen years of age. They were visited in the course of the evening by their friend, the well known Dr. Ruge, professor of German in Brighton, who found them playing at chess together. He took tea with them, and in the course of the evening, during the occasional absence of the young man, Dr. Franck referred to him in the most affectionate manner, praising him highly for his studies, and looking forward to his success in his profession. Both father and son

* London, 1855.

were perfectly cheerful, and on the best of terms; and not a circumstance could afterwards be remembered by Dr. Ruge which would give rise to the slightest suspicion or apprehension of the dreadful deeds that were so shortly afterwards committed.

Dr. Franck was a native of Breslau, in Silesia, and one of the most respectable and learned men in Germany. He was well known in Berlin, where he moved in the first circles, being an intimate acquaintance of Baron Von Humboldt, Varnhagen Von Ense, and Chevalier Bunsen. He had a world-wide reputation among all the men of literary and scientific celebrity in Europe. He was formerly editor of the well-known *Allegemeine Zeitung*, at Leipsic, where he had lately resided. His father was a banker, and the deceased himself was very wealthy. About eighteen years ago he was married, at Rome, to a daughter of Prince Henry of Prussia, who died about ten years ago, leaving an only son, Hugo. When this latter had reached the age of thirteen he expressed a desire to enter the English navy, but his father wished him to remain until he was older. Six months ago, the son keeping to his original wish, the father brought him to England, and placed him at Dr. Burney's naval academy, at Gosport; he himself taking up his abode at Portsmouth. It appears that, in consequence of being over age there was a difficulty in getting the young man into the Royal Navy, and steps were consequently taken by his father to enter him at Mr. Green's, at Blackwall, his first voyage being to India, and the ship being fixed to sail in December next. The intermediate time, it was resolved, should be spent by Dr. Franck and his son with his intimate friend and countryman Dr. Ruge. Hence their arrival at Brighton on Friday afternoon, as above mentioned, where, preparatory to taking up their quarters at Dr. Ruge's, they took apartments at the Albion Hotel.

Dr. Ruge left the hotel about eleven o'clock, the young man having gone to bed about half an hour before, having with him a volume of Bulwer's novels. After Dr. Ruge's departure, Dr. Franck retired to rest, occupying with his son a double-bedded sleeping apartment at the loftiest part of the hotel. Nothing to attract attention occurred between this time and a quarter to six o'clock on the following morning, when a servant was alarmed by the crash of some falling body on the outside of the hotel, and on looking out of her bedroom window she perceived the body of a man in the area below. She immediately gave an alarm; some militiamen billeted in the hotel went out, and picked up the body, which was recognised as that of Dr. Franck. Life was found to be extinct. The large bone of the hip was completely crushed, and the bleeding from the ruptured vessels was excessive.

Dr. Carter proceeded up stairs, where the lad was supposed to be asleep, and knocked at the bedroom door. No response was given to repeated knocking and shouting. The door was then broken open, and there lay the son, strangled in his bed. Life was extinct, though the body was still warm. A silk handkerchief was found lightly tied round his neck, but not, to all appearance, in such a manner as to have been the instrument of death; nor was there any appearance of a struggle having taken place in the bed or the room. The younger deceased was in his night clothes. The father had on slippers, drawers, a shirt, and dress coat, but no trowsers. His last act before going to bed was to ask for a newspaper, a copy of which was found by the side of the bed. He had previously asked for a candle, doubtless for the purpose of reading the paper. It is stated that in the course of the evening, while the two deceased were playing at chess, the waiter was struck by the peculiar manner in which Dr. Franck looked at his son when the young man made a move. This is the only thing that can be recalled by the waiter as having excited his particular notice.

The inquest was held at one o'clock on Saturday, at the Albion Hotel, before Mr. D. Black, coroner for the borough. In addition to the above facts, which were elicited during the inquiry, the following evidence was adduced:—

Dr. Arnold Ruge deposed—I have been acquainted with the elder deceased since 1840. He was about fifty-four years of age, and was a doctor of philosophy. I knew the son when he was a child, in Dresden and Berlin. The father was in easy circumstances. He at all times appeared sane, and of good understanding. He was a very sharp critic and a learned man, and he numbered amongst his friends all the learned men of his country. The boy yesterday said he thought he should go to sea about December. He said he still wished to go; he was quite determined upon that. There was no conversation with a view to persuade the boy not to go to sea. I asked the boy respecting his prospects, and he said he was determined to go to sea. The father and son appeared to be on the most affectionate terms. The father had been ill; yesterday he seemed in his usual health. He looked better, and was in a better humour than ever before. There was nothing in the conversation to induce me to contemplate the fearful result which followed. The mother died about ten years ago. As far as I know, the elder deceased had suffered no loss in fortune; I think not. The father said it was an unusual determination for a boy in Germany to wish to go to sea, and that it was very difficult for him to part from his son, as you will see by his letters. The father spoke on other indifferent subjects. Nothing was said of a desponding character, except of his son's attachment to the sea. The son bade his father good night in a most affectionate way. It did not appear that the son wished to go to sea in opposition to his father. Although the father did not wish it, there was nothing like opposition to it. The son was fond of horses and ships, and the father preferred ships to horses. The father's general demeanour was calm. I have never seen him excited. At the time of the movement in 1848, for instance, he was exceedingly calm. Although of the Democratic party, he took no part in the movement. His health was generally bad. He did not complain of a goitre, or large swelling, which he had in his throat. He kept it a secret. I don't know that the swelling affects the general health. The natives of Germany are very subject to it.

One or two of the hotel attendants having been examined with respect to the finding of deceased in the area, &c.,

Dr. Carter was called; and having deposed to the nature of the injuries inflicted, and other matters above referred to, continued in reply to various questions:—On the door of the younger deceased's bedroom being burst open, I found the young man lying on his back in the bed. I detected a silk scarf tied round his neck. Life was extinct. I immediately examined the state of the bed-clothes, which were unruffled. The posture of deceased showed no evidence whatever of a struggle, and I was particularly impressed with the fact that the silk scarf was so lightly tied round the neck, that I do not believe, as a surgeon, the stricture so caused could have produced death.* This necessitated the tightening of the scarf, if it were suicide. For many years the possibility of self-strangulation was denied; and I cannot, on the spur of the moment, remember but one such case, and that a disputed one. Pichegru is said to have been murdered, because he was found strangled, as was said, by his own hands, with ligature and a stick. It was denied that he could so have produced death, which would have been far easier than in this case. The determined Greenacre failed in self-strangulation. On the other hand, the whole history of the life of the father—his affection for his son, his strong mind, his strong principles—really seem to prove the improbability of his having destroyed his own son; but they equally point to the impossibility of his having destroyed himself. Looking at the state of health of the father; bearing in mind that he suffered habitually from dyspepsia—a most depressing disease; seeing, likewise, that he presented an enormous goitre in his throat, which was so large that it must have pressed considerably upon the blood vessels, and might have so disturbed the circulation of the brain; remembering that parting from his son appears ever to have been most distasteful to him, I think it more reasonable to suppose that the father, whilst under the influence of a temporary attack of insanity from those causes, should have decided, not only on stopping his son going to another hemisphere, but upon destroying his own life and his son's at one and the same time, that they might die together and not be separated. I can conceive that, with the cunning peculiar to such a homicidal idea, he should have crept out of bed whilst his dear son lay sleeping; that he should have passed the scarf without awaking him under his neck. So learned a man would be most likely to understand sufficient of physiology to know that slight pressure upon the surface veins, by retarding the flow of venous blood from the brain, and stopping the efflux of arterial blood to it, would have the effect of poisoning the brain with impure or carbonized blood. He would know that, without pain and without a struggle, he could so convert the sleep of fatigue into the sleep of death—a death as easy and similar to that produced by burning charcoal in a close chamber. I believe this explanation to be far more likely to be correct than that a youth in the heyday of life, about to join a profession he delighted in, should have committed suicide, and that his father should have done the same at the same moment in consequence of it. The handkerchief was tied very firmly indeed in a common sort of knot. I could get my two fingers in it. Pulling the ends would not tighten the knot. I don't think the son had been dead but a very few minutes. I believe the father and son died nearly together.

Police-officer Blaber deposed that he found two gold watches lying on the dressing-table, and three sovereigns, 11s. 9d., and a Bank of England note. He found in one of the portmanteaus a purse containing £8 10s. in gold, some clothes, books, and other things. The window was wide open.

The Coroner, whilst allowing full force to the opinion of Dr. Carter, held it to be improbable for a scarf to be inserted round the neck of the young man, in the heyday of youth, as it had been stated, and at six in the morning, with-

* It appears from a letter published in *The Times* that the youth had been in the habit of going to bed with a silk scarf round his neck.

out waking him. He inclined to the belief that the son, who, perhaps from reading, had been induced to adopt the sea as a profession, but who, from being brought into contact with the sailors at Portsmouth, had taken a disgust to the choice so long adhered to, and being too proud to confess his change of mind to his parent, might have perpetrated the horrid deed of suicide to get himself out of the difficulty. There was nothing improbable, also, in believing that the father, being a fond parent, should have committed the act of self-destruction immediately on discovering the position of his son.

The jury retired, and after an absence of about ten minutes, returned as their verdict—"That the son was found strangled in bed, but whether by his own hands, or by the hands of another, there was no evidence to prove; that the father destroyed himself, by throwing himself from the window, whilst labouring under an unsound state of mind."

The questions for the consideration of the jurist are as follows: was this a case of self-strangulation, the father destroying himself after discovering the fact of his son's suicide? or did Dr. Franck strangle his son, in a fit of homicidal insanity, whilst he was sleeping, and afterwards destroy himself? or did the son die suddenly from disease of the heart or brain, and the father, in a paroxysm of delirium brought on by the discovery of the fact, precipitate himself from the window of his bed-room? It will be well, before discussing these various points, to obtain, if possible, some insight into the constitution of Dr. Franck's mind. Mr. Henry Reeve, in his interesting letter to *The Times*, affords us some valuable information on the point.

"To those who were personally acquainted with this unfortunate gentleman (and in the number of his friends I might reckon no inconsiderable number of the first names in Germany and in other parts of Europe) it would be needless to repeat that the whole tenour of his life and character rebuts in the very strongest manner the suspicion of even an involuntary crime. He was a man of singularly clear and calm judgment, methodical and temperate in his habits, quiet and refined in his manners, endowed with the nicest sense of propriety and honour, of strong reasoning powers, often playful and humorous in his mode of expressing his opinions, and free from all the influences which commonly disturb the passions or distort the mind. His most absorbing interest in life was the education and future welfare of his son, to whom he was devoted with more than paternal tenderness; and, as an example of the affectionate care with which he watched over him, I am informed that it had been his practice to cause the boy from infancy to occupy a separate bed in his own sleeping apartment. This circumstance has proved, by an extraordinary fatality, one of the causes which aggravate this inexplicable catastrophe by some suspicions of madness or even of guilt. Dr. Franck has unhappily perished in a manner the most sudden and the most awful; though it is impossible to conceive that he was a voluntary agent in this scene of horror, or that he could have violated all the strongest feelings of his nature by the murder of his child. But neither can any traces be discovered, as far as I am informed, of the slightest tendency to insanity in his former life or conduct. He spent the previous evening with his son and Dr. Ruge in games of chess and tranquil conversation. No trace of a difference had been observed between himself and his son; and, although he had undoubtedly regretted and opposed the strong desire of the lad to go to sea when first that desire was manifested, yet he had often expressed to myself and other friends his conviction that it was better to gratify than to thwart so decided an inclination, and he had come to this country solely for the purpose of assisting in the fulfilment of this project. In one of the last letters addressed by Dr. Franck to the oldest of his friends in this country, on the 17th of October, the following passage occurs with reference to the measures which had been taken in concert with some members of her Majesty's Government to enable his son to enter the Royal navy — an object which he had much at heart. His words (written by himself in English) are deeply touching when contrasted with the terrible event which has blasted so much affection and so much hope. He wrote,—

" 'Do not wonder at the singular importance I attach to this matter — (the admission of his son into the Royal navy). I am sure you will have remarked a great deal of concern about me with respect to it; but nothing is more pardonable, and I dare even say more justified by circumstances. If I say that he is one of the rarest specimens of a boy, I am by no means afraid to be under any paternal illusions, for I repeat only what everybody who has the opportunity of observing him says, and what Mr. Burney (his master) more than once declared and lately wrote about him. To do my best that he may be placed in an adequate condition is an impulse of an aggregate of feelings for which *duty* is a poor word. I was troubled as long as I was kept uncertain whether I should be able to do really my best; now, as I have succeeded in this, and even far better than I expected, I feel comfortable, because I cannot pretend to have the command of the result.' "

In a letter which *The Times* did us the honour to publish in relation to this mysterious tragedy, we entered somewhat at length into this subject. We make no apology for reprinting a portion of this communication in our " Quarterly Psychological Retrospect."

" There appears to have been no assignable motive for the suicide of the son. Admitting that there was a prior unrecognised state of morbid mental depression, likely to originate a suicidal idea, the question is, could the son strangle himself in bed by tying a handkerchief with sufficient tightness round his throat to effectually impede the circulation of the vessels of the neck, or obstruct the act of respiration? Is this possible? There are too many instances of voluntary strangulation upon record to make the matter at all doubtful. General Pichegru's case is one remarkably in point. He was found strangled in prison during the consulate of Buonaparte. The body was found lying in the bed on the left side in an easy attitude, with the knees bent, and the arms lying down by the side. A black silk handkerchief was found twisted tightly round the neck by means of a stick passed under it. The cheek was torn by the ends of the stick during its rotations. There was no doubt of the case being one of voluntary strangulation. The Malays frequently commit suicide in the same way; cases of a similar character have come under my own observation. Was Dr. Franck's son a case of voluntary strangulation? There is no evidence of the fact. The loose condition of the handkerchief, the appearance of the countenance, the absence of all symptoms of a ligature having been applied round the neck, according to my judgment, is strong, if not demonstrative evidence of the son's death not being self-inflicted. Did the son die a natural but a sudden death, caused by disease of the heart, or did he expire during a fit of apoplexy or of epileptic convulsions? If such was the case, it would not be difficult to realize the terrible agony of the fond father upon making the sad discovery, and one can well conceive that the severe shock he would experience might give rise to temporary suicidal delirium. It will be impossible to settle the first point in the absence of a post-mortem examination. The heart, upon investigation, may be found organically diseased; if so, the death is easily accounted for. The state of the bed-clothes and the position of the body forbid altogether the supposition of a struggle having taken place between the father and his son. If he had died in a convulsive fit, the bed-clothes, the physiognomy, and state of the tongue would have indicated the fact. Again, death may have been caused by apoplexy without convulsions; but this is an unusual mode of death at so early an age, unless the heart is at the same time extensively diseased. A post-mortem examination of the brain could alone solve the point. If the son did not die by his own hands, or suddenly of disease of the heart or brain, what was the cause of his death? I repeat, that I do not believe that Dr. Franck, in the possession of his right mind, murdered his son. It is possible, I grant, that in a sudden paroxysm of homicidal insanity he may have done so. If this supposition is at all tenable, there would have been some signs of the struggle that must have ensued. It is very improbable that the father, in a paroxysm of suddenly-developed insanity, could have murdered his son without a fearful and hard-fought struggle for life having taken place. Again, setting aside altogether the hypothesis of insanity, may not the father have rushed upon and murdered his son while under the influence of a nocturnal vision, or some horrid phantasy originating during a troubled dream, and, while in this state of mind, have walked out of the window, or even have thrown himself from the window after being restored to healthy consciousness? Persons have been known to commit murder while in a state of somnambulism, or sleep-walking, and also during the half-unconscious condition between sleeping and waking. A person has been suddenly roused by a frightful dream, and while under its influence has been known to take away human life. Suicide has been committed under analogous circumstances. A person apparently well has gone to bed without manifesting the slightest tendency to self-destruction; he awoke suddenly and destroyed himself. An old lady residing in London awoke in the middle of the night, went down stairs, and threw herself into a cistern of water, where she was found drowned. It is supposed that the suicide was the result of certain mental impressions conjured up in the mind during a dream. Dr. Pagan refers to the following interesting case, to prove that murder may be committed by a person when under the effects of a frightful vision:—Bernard Schedmaizig suddenly awoke at midnight. At the moment he saw a frightful phantom, or what his imagination represented as such—a fearful spectre. He twice called out, "Who is that?" and receiving no answer, and imagining that the phantom was advancing upon him, and having altogether lost his self-possession, he raised a hatchet which was beside him and attacked the spectre, and it was found, alas! that he had murdered his wife. A pedler, who was in the habit of walking about the country armed with a sword-stick, was awakened one evening while lying asleep on the high road by a man suddenly seizing him and shaking him by the shoulders. The man, who was walking by with some companions, had done this jocosely. The pedler, suddenly roused from his sleep, drew his sword and stabbed the man, who soon afterwards died from the effects of the wound. He was tried for manslaughter. His irresponsibility was strongly urged by his counsel, on the ground that he could not have been conscious of his act in the half-waking state. He was, however, found guilty, and subjected to imprisonment.

I am inclined to believe, from an attentive consideration of the facts of the distressing and remarkable case under review, that Dr. Franck's son died a natural, but a sudden death, and that if the body were exhumed and a post-mortem examination instituted, such would be found to be the fact. The father, I think, destroyed himself while in a paroxysm of temporary delirium, frenzy, or mental aberration, induced by the mental shock consequent upon the appalling discovery of his son's awfully sudden death.

Nothing that has subsequently been published on the subject has, in the slightest degree, altered our opinion as to the cause of death. We believe that Dr. Franck's son died suddenly, and it is quite reasonable to imagine that during the excitement and anguish which would necessarily arise on its discovery, the wretched father, in his anxiety to make the fact known in the hotel, and to obtain assistance, *either mistook, in the dark, the window for the door, and accidentally fell into the street, or, in a paroxysm of delirium, threw himself from the window.*

Is this an improbable occurrence? Dr. Hall, in an account of an examination he made of the body of the son some time after death and published in *The Lancet*, alleges that he discovered obvious marks of violence on the neck of the son; but are these alleged marks of finger-nails, detected some period after death, and subsequently to putrefaction having taken place, of any scientific value? We think not. If these marks had existed when Dr. Carter saw and examined the body, he certainly would have deposed to them when giving evidence at the Coroner's inquest. The hypothesis of sudden death is greatly strengthened by the important fact referred to in the subjoined extract from a letter written by a physician resident at Berlin:—

"Hermann Franck lived in high life, and in easy circumstances. By a long sojourn in England, France, and Italy, he had acquired a profound knowledge of the treasures of art and literature of those countries. He is well known as a public character with us; he was a man of kind feelings and the most refined education — beloved and esteemed by Varnhagen Von Ense, by Humboldt, by Bunsen, an intimate friend of Felix Mendelssohn, and of my brother.

"*The early death of his wife had made him look upon his son as the only earthly blessing left to him. He loved, he adored the youth; he watched him with anxiety, because the boy, being the very image of his mother, who suddenly died from a disease of the heart, showed apparent symptoms of that disease.*

"What is now more likely to be supposed, but that the youth, excited by the decisive events of the last days, died by a similar disease? Who can think, in this case, of suicide of the boy, which, indeed, is not admitted by the surgeon at the inquest; or of murder, which the coroner declined to believe, and the jury did not think proper to decide upon?

"The corpse of the youth lay in bed like a sleeping one. The handkerchief round his neck was not drawn tight enough to produce death. It is to be regretted that in the English reports nothing is to be found respecting the dissection of the body.

"The frightful aspect of his destroyed son drove the poor father to despair, and he finished his life by throwing himself out of the window."

The Coroner has been greatly censured for neglecting to order a post-mortem examination of the body. In order to ascertain the cause of death under the suspicious circumstances accompanying the decease of Dr. Franck, a searching post-mortem examination should have been instituted into the state of the heart and brain, and the contents of the stomach ought to have been subjected to a chemical analysis, with the view of ascertaining whether poison had been taken. A case of this grave importance should not have been left enshrouded in doubt and mystery, when the scalpel of the morbid anatomist could so easily have solved the question as to the true cause of death.

It was our intention to print in detail the report of the important trial to which the subjoined criticism refers, but want of space compels us to abandon the idea. The animadversions of our able and acute contemporary, *The Lancet*, are so pertinent and just, that we should only weaken the case if we were to add any comment of our own on the subject.

"A recent case, heard before Mr. Justice Erle, strikingly exemplifies the confusion that reigns in our legal treatment of insanity. A man possessed with the belief that his wife's children were not his own, but begotten by a clergyman, threatened repeatedly that he would shoot the latter in his pulpit. Nor was his threat an unmeaning one. He went to the church, provided with percussion-caps, Minié balls, and, as he thought, a Minié pistol in a bag; but he had by a fortunate mistake taken up the wrong—or we ought rather to say the right—bag, leaving behind at his hotel that which contained the pistol. That he was acting under an insane delusion—that there was no ground for his belief, is fully admitted. The clergyman very properly applies to the law for protection. Mr. Justice Erle expounds the law, and the result is this: The Judge starts by saying that the defendant is evidently '*more diseased than guilty;*' but since he is not proved to have more than *this one delusion*, it would not be right to take him away from his wife and family, or to restrain his liberty; he *therefore* decides that this man, more diseased than guilty, is to enter into his own recognizances to keep the peace towards the clergyman whom he had sought to shoot in his pulpit! and *if he should threaten again*, that he should be brought before the Court to receive judgment!!

"Will he *threaten* again? Is not it quite as probable that he will carry his design into execution without saying a word more about it? Mr. Justice Erle, no doubt, thinks he will not; for Mr. Justice Erle is of opinion that, although the defendant was more diseased than guilty, his disease was so essentially a moral and not a physical one, that it was to be cured by a judicial admonition administered from the bench! His lordship solemnly appealed to the delusionist to dismiss his delusion from his mind, and not to threaten or molest the clergyman any more. We sincerely hope, for the clergyman's sake, that this admonition will have a curative effect; and if it should, it will then become a question calling for the most serious consideration, whether Mr. Justice Erle should not receive her Majesty's commission to visit all the lunatic asylums, to administer judicial exhortations to madmen to give up their delusions—in short, to order them to cure themselves, and on the strength of their assurances and recognizances to discharge them! But we fear it may happen that this admonition may not prove curative, and that the next intimation of the persistence of the defendant's delusion may be the homicide of its object.

"What will be the logical conclusion from this issue of the practice of treating insanity in courts of justice? The madman will of course be put upon his trial for murder. We will suppose that Mr. Justice Erle is again his judge. What will be his lordship's judgment when he finds that his admonition has failed? Will he still regard him as guilty as well as diseased? Will he punish the guilt notwithstanding the disease? Will he sentence him to be hanged? Or will he try the effect of another admonition? Or will he, taught by his little success in the cure of insanity, regard the disease as the cause of the homicide, that this causation excludes the idea of guilt, and come to the conclusion that the physician would do more good than hangman or judge?

"Yet this case is but the practical application of the views advocated by Dr. Mayo, which confound insanity and sin, by maintaining the possibility of their simultaneous existence in the same person, by making the man more diseased than guilty responsible alike for his disease and its consequences. It was this doctrine that led to the judicial murder of Buranelli, and which, if carried out logically, will in all likelihood end in the homicide of a clergyman and the legal murder of another madman."

With the preceding able comments upon a case having an important medico-legal bearing we close our first Quarterly Psychological Retrospect. There were other topics of deep psychological import to which it was our intention to have adverted, but the Retrospect, having already considerably exceeded the prescribed limits, must be brought to a conclusion. We hope for the future to make this department of the Journal one of its most important and interesting features. It will be our object to collect from various sources, foreign as well as British, the salient facts which, according to our judgment, may be considered illustrative of the psychological history of the times. In this busy world, and during the eventful epoch in which we live, events of an important nature are daily occurring necessarily involving in their consideration and discussion questions of a psychological character. These matters will constitute in the main the basis of our future Quarterly Psychological Retrospect.

23, *Cavendish Square,*
*January* 1, 1856.

